# Supplementary material for: The Dominant factors and optimization plan of soccer substitutions in Chinese Football Association Super League under the Five-substitution rule
Source: PLoS One. 2025 Jun 23;20(6):e0326656. doi: 10.1371/journal.pone.0326656 (PMC12185027; doi:10.1371/journal.pone.0326656)
Supplement: Attachment — Submitted filename: Response to Reviewers.docx [file pone.0326656.s006.docx]

Dear Academic Editor,

Thank you for giving us the opportunity to submit a revised draft of our manuscript titled "The Dominant factors and optimization plan of soccer substitutions in Chinese Football Association Super League under the Five-substitution rule" [PONE-D-25-18355] to PLOS ONE. We appreciate the time and effort that you and the reviewers have dedicated to providing valuable feedback on our manuscript. We have incorporated changes that reflect all the suggestions provided by the reviewers. All changes are highlighted in red in the revised manuscript. Please see below for our point-by-point responses to the reviewers' comments. We hope that our work can be improved again.

**Response to Comments of Reviewer *#*1：**

**Comment 1**：As demonstrated in Table 2, the current form of the table is rather dense and challenging for readers to comprehend. The presentation of a large number of variables and test results together has been shown to reduce the readability of the table. Consequently, the table should be restructured to be more straightforward and lucid, with emphasis placed exclusively on statistically significant and interpretation-critical variables.

**Response to comment 1:** We appreciate this thoughtful suggestion. Upon review of Table 2, we acknowledge that the presentation of numerous variables could reduce its clarity. As you pointed out, one variable lacked statistical significance, and we have therefore removed it from the table.

The revised Table 2 now exclusively includes those variables that demonstrate statistical significance. In addition, we only described statistically significant variables in the results section. This change enhances the clarity of both the table and the overall discussion of the results.

**Comment 2**：The Conclusion section is largely a reiteration of the Discussion section. The conclusion should summarise the main findings of the study in a concise, original and striking way; it should also include possible application areas or suggestions for future studies.

**Response to comment 2:** We agree with your observation and have rewritten the Conclusion section to present the core findings in a more concise and original manner, highlight the practical implications of the study for football performance analysis, and provide possible application areas based on our results.

**Comment 3:**The employment of language can, on occasion, be somewhat lacking in fluency, with the result that ambiguity may be introduced into technical expressions. It has been observed that certain sentences appear to exert a translation effect. It is imperative that the language of the article be revised in order to achieve a more academic and natural English style. This process should be undertaken by an expert whose native language is English.

**Response to comment 3:** Thank you for pointing this out. To address this, we have had the entire manuscript professionally edited by a native English-speaking professor. The revised version reflects improvements in fluency, clarity, and academic tone. We trust this resolves the language issues previously identified.

**Response to Comments of Reviewer *#2*:**

**Comment 1:** Please consider refining the subheadings within the Results and Discussion section to improve clarity and logical flow.

**Response to comment 1:** We appreciate this suggestion. We have revised the subheadings throughout the Results and Discussion section to better reflect the thematic structure and logical progression of our analysis. This improves navigation and enhances the reader’s understanding.

**Comment 2:** Ensure that all abbreviations are introduced with their full terms upon first mention to facilitate understanding.

**Response to comment 2:** We carefully reviewed the entire manuscript and ensured that all abbreviations are now introduced with their full terms upon first use to assist the reader.

**Comment 3:**Review the overall formatting for consistency and alignment with academic presentation standards.

**Response to comment 3:** We conducted a thorough formatting review of the manuscript. The revised version aligns with the journal’s style guide in terms of headings, references, table and figure formatting, and general presentation standards. We have also re-checked the layout for consistency.

Think you and best regards.

Yours sincerely

CHEN Tong

Corresponding author:

Name: CHEN Liang

E-mail: cullencl@126.com
